# Early Surgery Reduces Infection Risk and Length of Hospital Stay in Closed Ankle Fractures: A Retrospective Cohort Study

**DOI:** 10.3390/jcm14176161

**Published:** 2025-08-31

**Authors:** Roberta Laggner, Cornelia Gärtner, Emily Ghanbari, Florian Bur, Michael Humenberger, Thomas Haider

**Affiliations:** 1 Department of Orthopedics and Trauma Surgery, Medical University of Vienna, 1090 Vienna, Austria; n0543698@students.meduniwien.ac.at (C.G.); florian.bur@meduniwien.ac.at (F.B.); michale.humenberger@meduniwien.ac.at (M.H.); thomas.a.haider@meduniwien.ac.at (T.H.); 2 Department of Cardiology, Angiology and Intensive Care, German Heart Center of Charité, Campus Benjamin Franklin, 12203 Berlin, Germany; emily.ghanbari@charite.de; 3 Institute of Active Polymers, Berlin-Brandenburg Center for Regenerative Therapies, Helmholtz-Zentrum Hereon, 14513 Teltow, Germany

**Keywords:** ankle fracture, surgical timing, postoperative infection, surgical site infection (SSI), fracture fixation

## Abstract

**Background:** The optimal timing of surgical treatment for ankle fractures remains a topic that is associated with clinical uncertainty. While delayed surgery is often necessary for safe wound closure, prolonged immobilization, impaired functional outcomes, protracted hospitalization, and an increased risk of infection are potential disadvantages. This study was aimed at investigating the interval between trauma, surgical fixation, and postoperative infections among patients with closed ankle fractures. **Methods:** We conducted a retrospective cohort study involving 224 patients treated surgically for fractures of the upper ankle joint between January 2020 and December 2023. The patients were stratified into two groups based on surgical timing: within 24 h of hospital admission (early surgery) or after 24 h (delayed surgery). The primary outcome was the incidence of postoperative infections. A multivariate logistic regression model was constructed to assess independent risk factors. **Results:** Of the 224 patients, 30 (13.4%) developed postoperative infections. Infection occurred in 11.1% of patients who underwent early surgery and 13.7% of those subjected to delayed surgery. This difference was not statistically significant in the unadjusted analysis (*p* = 0.747). However, an additional day of surgical delay was associated with an 11% increase in the odds of postoperative infection (OR = 1.11; 95% CI: 1.01–1.22; *p* = 0.034). Female patients had over threefold higher odds of infection than males (OR = 3.20; 95% CI: 1.32–8.09; *p* = 0.011), and diabetes was a significant risk factor, with diabetic individuals showing more than fivefold increased odds (OR = 5.56; 95% CI: 1.30–25.00; *p* = 0.019). Patients with delayed surgery had significantly longer hospital stays (+2.83 days, *p* < 0.05). **Conclusions:** Early surgical intervention appears to lower the risk of postoperative infections, is associated with hospitalization duration, and should be considered when clinically appropriate.

## 1. Introduction

Ankle fractures represent one of the most frequent orthopedic trauma injuries, accounting for up to 10% of all skeletal injuries. Their incidence has steadily increased over recent decades, a trend largely attributed to demographic shifts and increased physical activity among the aging population [1,2,3]. While many simple fractures can be managed conservatively, 30% to 70% of ankle fractures require surgical intervention [4,5]. The most frequent early complications of operative treatment are surgical site infections (SSIs) and wound-healing disorders. Reported SSI rates after open reduction and internal fixation (ORIF) vary widely across studies, from 1.5% to 17% in general populations [6,7]. The rate of deep infections varies from 2.8% to 6.8% for [8,9].

SSI has a considerable impact on patient outcomes and health system costs. It is associated with impaired functional recovery, increased complication rates, and significantly longer hospital stays [10]. The economic burden is also substantial, as SSIs lead to 3- to 8-fold higher hospital costs due to prolonged hospital stay, antibiotic therapy, and revision surgeries [11].

Despite these implications, the optimal timing of ankle fracture surgery remains a matter of controversy, particularly regarding the risk of postoperative infection. Delayed surgery with improved soft tissue conditions reduces complications, whereas early fixation is often preferred to minimize hospital stay and accelerate mobilization. On the other hand, delayed surgery may increase the risk of SSIs, particularly if tension blisters appear, which can lead to bacterial growth. Additionally, delayed surgery does not always result in a sufficient reduction in swelling.

In this study, we aimed to evaluate whether surgical timing influences postoperative infection rates and length of hospital stay and identify patient- and injury-related predictors of infection using multivariate analysis.

## 2. Materials and Methods

A retrospective cohort study was conducted at a Level I trauma center, including adult patients (aged ≥18 and <80 years) who underwent surgical treatment for fractures of the upper ankle joint between January 2020 and December 2023. The exclusion criteria were polytrauma, open fractures, prior local infections, prior management with an external fixator, incomplete documentation, or infrequently used surgical approaches (e.g., posterolateral or posteromedial approach). Postoperative infections were defined based on clinical presentation and classified according to Centers for Disease Control and Prevention (CDC) criteria, as adapted from Schepers et al. [12]. This approach includes both superficial and deep infections, identified through clinical signs and the need for antibiotic therapy or surgical intervention. We note that this definition differs from the more recent fracture-related infection (FRI) consensus criteria. As such, direct comparisons with studies using FRI-based definitions should be made with caution.

Data collection included demographic variables (age, sex), timing of surgery, length of hospital stay, and postoperative infections. Fracture complexity was classified using a combined scheme based on Weber classification and the number of malleoli involved: low (unimalleolar or Weber A), moderate (bimalleolar or Weber B), and high (trimalleolar or Weber C).

Statistics: The primary outcome was the occurrence of postoperative infections, as determined by clinical findings, antibiotic administration, revision surgery, or wound healing disorders. Statistical analyses included descriptive statistics and comparisons using chi-square or *t*-tests, as appropriate. A multivariate logistic regression model was implemented to identify independent predictors of postoperative infections. The predictor variables included age, sex, comorbidities, surgical delay, and fracture complexity. Odds ratios (ORs) with 95% confidence intervals were calculated. A Cox model was fitted using the time from surgery to infection as the survival time. The outcome variable was defined as the occurrence of a postoperative infection. The model was adjusted for clinically relevant covariates, including the ASA (the American Society of Anesthesiologists physical status classification system) score (I–III), age, diabetes, fracture classification according to Weber (A, B, C), fracture mechanism (low- vs. high-energy trauma), and fracture type (closed vs. open). Cox models were built using the coxph() function in R, reported with 95% confidence intervals. Statistical analyses were conducted with SPSS Statistics (SPSS 24.0 for MAC, IBMCorp., Armonk, NY, USA) and R software (Version 4.3.3). An adjusted *p*-value of *p* < 0.05 was considered statistically significant.

Ethics: The local institutional review board approved the study protocol before data collection and waived the need for individual informed consent. All patient records were anonymized and de-identified prior to analysis. This study was performed according to the Declaration of Helsinki, the ICH Harmonized Tripartite Guideline for Good Clinical Practice, and the guidelines of the local Institutional Review Board.

## 3. Results

A total of 224 patients underwent surgical treatment for closed ankle fractures and were included in the final analysis. The mean age was 47.8 years (range: 18–80), with 63.4% (*n* = 142) identifying as female. Female patients were, on average, older than males (mean 50.6 vs. 42.9 years), with a mean difference of 7.7 years. Table 1 summarizes all baseline characteristics.

Fracture Pattern: Fracture laterality was evenly distributed (left vs. right), with no significant difference between early and delayed surgery groups (*p* = 0.966). Weber B fractures were most prevalent (67.9%), followed by Weber C (29.0%) and Weber A (3.1%). Based on malleolar involvement, 35.3% were unimalleolar, 50.4% bimalleolar, and 14.3% trimalleolar. While trimalleolar fractures had the highest infection rate (21.9%), this association was not statistically significant (χ^2^(2) = 2.753, *p* = 0.252). According to the AO/OTA classification, approximately 3% of the fractures corresponded to infrasyndesmotic types (AO 44-A), 68% to transsyndesmotic fractures (AO 44-B)—a substantial proportion of which were bi- or trimalleolar—and about 29% to suprasyndesmotic fractures (AO 44-C).

Infection: Postoperative infections occurred in 30 of the 224 patients (13.4%). According to CDC criteria adapted from Schepers et al. [12], 21 cases (70%) were classified as mild, requiring no intervention or only oral antibiotics. In comparison, nine cases (30%) were classified as severe, necessitating intravenous antibiotics, wound debridement, revision surgery, or implant removal. Of the 27 patients (12.1%) who underwent early surgery (<24 h), three (11.1%) developed infections—one mild and two severe. Among the 197 patients (87.9%) who underwent delayed surgery (≥24 h), 27 (13.7%) developed infections—20 mild and seven severe. Although severe infections were proportionally more frequent in the early surgery group, the minimal sample size in that group limits statistical interpretation.

Therapeutic Measures: Among the 30 patients who developed postoperative infections, oral antibiotic therapy was the most frequently administered treatment, given to 23 patients (76.7%). Intravenous antibiotics were required in five patients (16.7%), either as a primary measure or in addition to oral therapy. In 10 cases (33.3%), a surgical wound debridement was performed. Revision surgery—defined as any reoperation due to infection, including implant removal—was required in three patients (10.0%), of whom two underwent implant removal and one required additional surgical intervention. Furthermore, four patients (13.3%) exhibited signs of wound healing disturbances that resolved without the need for further intervention. While nine infections were classified as severe based on CDC-adapted criteria, 10 patients underwent debridement. This slight discrepancy reflects clinical decision-making: one patient received debridement despite not fulfilling all severity criteria and was, thus, categorized as having a mild infection.

**Figure 1:**
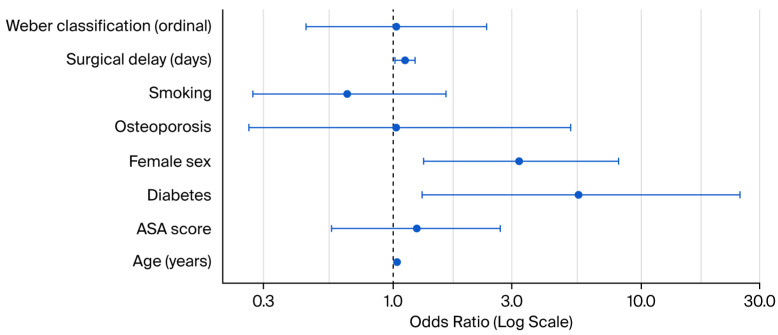
Logistic regression: effect of surgical delay and covariates on postoperative infections.

In the multivariable logistic regression analysis, several predictors were significantly associated with postoperative infection. Female sex was linked to a more than threefold increase in infection risk (OR = 3.20;95% CI: 1.32–8.09; *p* = 0.011), and each additional day of surgical delay was associated with higher odds of infection (OR = 1.11;95% CI: 1.01–1.22; *p* = 0.034). Diabetes emerged as a strong independent risk factor, with diabetic patients showing over five times the odds of infection compared to non-diabetics (OR = 5.56; 95% CI: 1.30–25.00; *p* = 0.019). Other variables, including age, ASA score, smoking status, osteoporosis, and fracture classification, were not significantly associated with infection risk (Table 2 and Figure 1).

**Figure 2:**
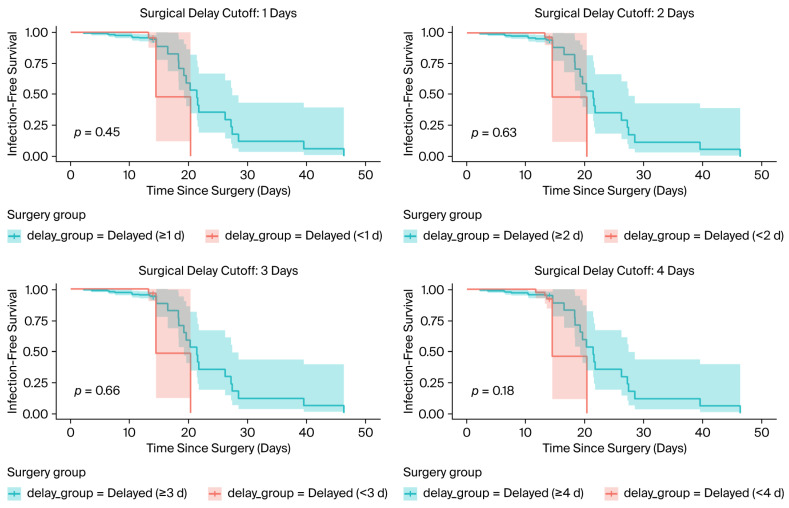
Kaplan–Meier survival analysis stratified by different delay thresholds (1 to 4 days).

To further explore time-dependent effects, Cox-Model and Kaplan–Meier survival analysis were performed for different surgical delay thresholds (1, 2, 3, and 4 days) (Figure 2). Across all cutoffs, there were no statistically significant differences in infection-free survival between early and delayed surgery groups, with log-rank *p*-values ranging from 0.18 to 0.66. However, visual separation of the curves emerged around 15–20 days post-surgery, and the wide confidence intervals and limited event count limited statistical power.

The time-to-event Cox regression model, using the same covariates, did not identify any statistically significant predictors of postoperative infection. Surgical delay (HR = 0.99; 95% CI: 0.91–1.07; *p* = 0.728), ASA score, age, fracture severity (ordinal Weber classification), and trauma mechanism all showed non-significant associations, with low overall model discrimination (concordance = 0.62) (Table 3).

Length of Hospital Stay: Patients who underwent surgery more than 24 h after admission had significantly longer hospital stays, averaging 2.83 additional days compared to those treated earlier (*p* < 0.05), highlighting a potential efficiency benefit of timely surgical intervention.

## 4. Discussion

This study comprises an investigation of the effect of surgical timing on postoperative infection in ankle fracture patients and an identification of key risk factors using multivariate analysis. Delayed surgery beyond 24 h was significantly associated with an increased risk of infection, and each additional day of delay raised infection odds by 11%. Although a daily 11% increase in odds may appear modest, cumulative delays of 3–5 days may result in clinically meaningful increases in infection rates. These findings indicate that timely surgical intervention—when clinically feasible—may help reduce postoperative complications. Additionally, diabetes and female sex emerged as strong independent predictors of infection, underscoring the need for individualized risk assessment.

The significant association between surgical delay and infection risk adds to an ongoing debate. While delayed surgery is often necessary to optimize soft tissue conditions, particularly in swollen or compromised extremities, this benefit must be balanced against the potential risk of infection. Our results align with the results of prior studies suggesting that prolonged preoperative intervals may increase susceptibility to infection, potentially due to factors such as prolonged immobilization, bacterial colonization, or more complex surgical courses. Although our dataset did not capture the specific reasons for surgical delays, it is plausible that in some cases, non-medical factors—such as limited operating room availability or reduced weekend staffing—may have contributed. Such logistical issues have been described in the literature and may represent modifiable barriers to early surgery in otherwise eligible patients [10]. Delayed surgery was also associated with significantly longer hospital stays, a pattern mirrored in several other studies. In clinical practice, surgical delays are not solely due to medical factors but often reflect logistical limitations, such as limited operating room availability or lower weekend staffing. Structured triage protocols and institutional prioritization strategies may help mitigate avoidable delays, particularly for closed fractures at high risk of infection [13].

Notably, our analysis did not identify fracture complexity as an independent risk factor. While complex fractures such as bimalleolar or trimalleolar types are often assumed to increase complication risk, this was not statistically evident in our cohort. As Tausendfreund et al. describe, fracture dislocation and open fractures remain more consistent predictors of infection, likely due to soft tissue compromise—factors less represented in our population of closed fractures [13,14].

Importantly, our study defined postoperative infection based on clinical presentation and the requirement for therapeutic intervention, such as antibiotic administration, wound debridement, or revision surgery—an approach consistent with common clinical practice. However, growing recognition of variability in infection diagnosis across studies has prompted calls for standardized definitions. In this context, Pilskog et al. applied the recently developed consensus definition of fracture-related infection (FRI), which incorporates both suggestive and confirmatory criteria, including microbiological, clinical, and radiographic findings [15]. In their cohort of over 1000 surgically treated ankle fracture patients, 9% met the FRI criteria for infection. Notably, 30% of suspected infection cases did not undergo adequate bacterial sampling, revealing a gap in systematic diagnostic work-up. This highlights a critical limitation in real-world practice and suggests that reliance on treatment-driven definitions, such as ours, may both under- and overestimate true infection rates. Future research should consider integrating the FRI consensus algorithm to enhance diagnostic precision and facilitate comparability across studies and institutions (15).

An unexpected but notable finding in our study was that female patients were more than three times as likely to develop postoperative infections compared to males. This contrasts with the content of earlier studies that identified male sex as a risk factor for surgical site infections (SSIs) in orthopedic trauma. For example, Dodd et al. analyzed over 6800 patients with ankle fractures, and they reported higher infection rates among males, potentially linked to higher-energy trauma mechanisms and behavioral risk factors such as smoking (7). Given this contradiction, our finding should be interpreted with caution. It may be influenced by residual confounding, chance variation due to the limited number of infection events, or characteristics specific to our patient cohort. This highlights inconsistencies in the literature and indicates a potential influence of sex-specific immune, vascular, or skin responses. Furthermore, hormonal influences on immune modulation—particularly estrogen’s role in inflammatory cytokine regulation—may alter the local and systemic response to surgical trauma. While speculative in the orthopedic context, such mechanisms warrant further investigation, particularly through prospective studies assessing sex-specific risk profiles in infection-prone procedures [16,17]. Diabetes, a well-established risk factor, was also strongly associated with infection in our cohort, reinforcing existing evidence on its detrimental effect on wound healing and immune defense [7,8,14].

Our two analytical approaches yielded different results. In the logistic regression, surgical delay, female sex, and diabetes were statistically significant predictors of postoperative infection. In contrast, the time-to-event Cox model—adjusting for the same covariates—did not identify any significant predictors. This difference arises because logistic regression considers whether an infection occurred at any point during follow-up, while the Cox model accounts for both the occurrence and timing of infection events. Given the relatively short and variable follow-up, the small number of infections, and the presence of right-censoring, the Cox model had limited statistical power to detect associations. This suggests that the absence of significance in the Cox model should not be interpreted as evidence of no effect. Instead, our findings highlight the importance of adequate follow-up duration and event counts in time-to-event analyses.

### 4.1. Limitations

This study includes a clearly defined cohort, standardized treatment protocols, and the use of multivariate modeling to account for confounders. However, its retrospective and single-center design limits generalizability. Additionally, a lack of intraoperative details—such as surgical duration or soft tissue status—may obscure other procedural risk factors described in the literature.

### 4.2. Implications for Practice and Research

Our findings support early but judicious surgical intervention, particularly for patients without substantial comorbidities, to optimize hospital efficiency without increasing infection risk. Risk stratification tools based on age, sex, ASA score, and comorbidities may help identify high-risk individuals who could benefit from intensified perioperative management. Further prospective, multicenter studies are needed to validate predictive models and refine prevention strategies, as encouraged by the recent review literature.

## 5. Conclusions

Delayed surgical treatment of closed ankle fractures was associated with an increased risk of infection and longer hospitalization. When soft tissue conditions permit, early surgery should be considered to optimize outcomes.

## Figures and Tables

**Table 1:** Baseline patient characteristics.

Variable	Value
*n*	224
Age	47.8 ± 17.2
Sex	
Male	141 (63%)
Female	82 (37%)
ASA Score	
ASA Score—1	72 (32%)
ASA Score—2	120 (54%)
ASA Score—3	31 (14%)
Weber Classification	
A	7 (3.1%)
B	152 (68%)
C	64 (29%)
Smoking	59 (27%)
Diabetes	13 (5.8%)
Osteoporosis	16 (7.2%)
Hospital Stay (d)	8.4 ± 5.6
Time until Surgery (d)	6.8 ± 4.3 (range: 0–23 days)

**Table 2:** Logistic regression: effect of surgical delay and covariates.

Variable	OR [95% CI]	*p*-Value
Weber classification	1.03 (0.44–2.37)	0.941
Surgical delay (days)	1.11 (1.01–1.22)	0.034
Smoking	0.65 (0.27–1.63)	0.349
Osteoporosis	1.02 (0.26–5.19)	0.975
Female sex	3.20 (1.32–8.09)	0.011
Diabetes	5.56 (1.30–25.00)	0.019
ASA score	1.24 (0.56–2.69)	0.594
Age (years)	1.03 (1.00–1.06)	0.106

**Table 3:** Cox regression model: effect of surgical delay and covariates.

Variable	HR [95% CI]	*p*-Value
Surgical delay (d)	0.99 (0.91–1.07)	0.728
ASA score	0.74 (0.38–1.41)	0.356
Age (years)	1.01 (0.99–1.04)	0.362
Weber classification	1.64 (0.75–3.58)	0.216
Fracture mechanism (high/low energy trauma)	1.19 (0.50–2.79)	0.696

## Data Availability

Data are available from the authors on reasonable request.

## Abbreviations

The following abbreviations are used in this manuscript:

**Abbreviation**	**Full Form/Description**
SSI	Surgical Site Infection
ORIF	Open Reduction and Internal Fixation
CDC	Centers for Disease Control and Prevention
OR	Odds Ratio
CI	Confidence Interval
ASA	American Society of Anesthesiologists (score)
AO/OTA	Arbeitsgemeinschaft für Osteosynthesefragen/Orthopaedic Trauma Association
HR	Hazard Ratio
SPSS	Statistical Package for the Social Sciences
MAC	Macintosh (Apple Computer Platform)
IBM	International Business Machines Corporation
FRI	Fracture-Related Infection
ICH	International Council for Harmonisation
GCP	Good Clinical Practice
